# Marbling-Net: A Novel Intelligent Framework for Pork Marbling Segmentation Using Images from Smartphones

**DOI:** 10.3390/s23115135

**Published:** 2023-05-28

**Authors:** Shufeng Zhang, Yuxi Chen, Weizhen Liu, Bang Liu, Xiang Zhou

**Affiliations:** 1 School of Computer Science and Artificial Intelligence, Wuhan University of Technology, Wuhan 430070, China; 2 Sanya Science and Education Innovation Park, Wuhan University of Technology, Sanya 572000, China; 3 Key Laboratory of Agricultural Animal Genetics, Breeding and Reproduction of Ministry of Education, College of Animal Science and Technology, Huazhong Agricultural University, Wuhan 430070, China; 4 Hubei Hongshan Laboratory, Wuhan 430070, China; 5 Shenzhen Institute of Nutrition and Health, Huazhong Agricultural University, Wuhan 430070, China; 6 Shenzhen Branch, Guangdong Laboratory for Lingnan Modern Agriculture, Genome Analysis Laboratory of the Ministry of Agriculture, Agricultural Genomics Institute at Shenzhen, Chinese Academy of Agricultural Sciences, Shenzhen 518038, China

**Keywords:** pork marbling, pork quality evaluation, image segmentation, patch-based training, context encoder network

## Abstract

Marbling characteristics are important traits for the genetic improvement of pork quality. Accurate marbling segmentation is the prerequisite for the quantification of these traits. However, the marbling targets are small and thin with dissimilar sizes and shapes and scattered in pork, complicating the segmentation task. Here, we proposed a deep learning-based pipeline, a shallow context encoder network (Marbling-Net) with the usage of patch-based training strategy and image up-sampling to accurately segment marbling regions from images of pork longissimus dorsi (LD) collected by smartphones. A total of 173 images of pork LD were acquired from different pigs and released as a pixel-wise annotation marbling dataset, the pork marbling dataset 2023 (PMD2023). The proposed pipeline achieved an IoU of 76.8%, a precision of 87.8%, a recall of 86.0%, and an F1-score of 86.9% on PMD2023, outperforming the state-of-art counterparts. The marbling ratios in 100 images of pork LD are highly correlated with marbling scores and intramuscular fat content measured by the spectrometer method ($R^{2}$ = 0.884 and 0.733, respectively), demonstrating the reliability of our method. The trained model could be deployed in mobile platforms to accurately quantify pork marbling characteristics, benefiting the pork quality breeding and meat industry.

## 1. Introduction

Pork contains a large amount of protein and nutrients and has become an everyday staple diet in many countries such as China, the United States, and Europe [1,2]. Increased pork consumption has led to a substantial demand for high-quality pork. Improving pork quality is essential to the entire pork chain, from meat science research, genetic breeding, and production to the retailer. The richness and spatial distribution of marbling are important meat quality traits since they are associated with flavor, taste, and juiciness [3,4,5]. Marbling characteristics collected from the longissimus dorsi (LD) muscle of pork carcasses are widely used for the genetic improvement in meat quality in pigs [6].

Pork marbling comprises white intramuscular fat (IMF) particles accumulating between muscle fiber bundles within the lean meat. Currently, visual scoring is the most preferred approach to quickly evaluate the marbling characteristics, which is usually conducted by comparing the meat and marbling standardized pictures by experts [7,8]. However, it relies on subjective assessment and can produce unreliable and inconsistent results. Chemical analysis is another routine approach, such as the Soxhlet method, which measures the intramuscular fat content of pork and indirectly reflects the spatial distribution of marbling [9]. Even though it has been used for many years, the disadvantages in chemical analysis are apparent; they are destructive, expensive, time-consuming, and unable to describe the visual appraisal of marbling distribution that is highly valued for marbling evaluation.

To tackle these limitations, spectroscopy and imaging techniques have been adopted for marbling analysis in recent years, such as near-infrared reflectance spectroscopy [10,11], nuclear magnetic resonance [12], hyperspectral imaging technology [13,14], ultrasonic imaging [15], and X-ray computed tomography [16]. Among them, the computer vision system is recognized as the most promising technique for quantitatively assessing the marbling characteristics since it can provide superior spatial distribution information of the marbling samples and is faster, non-destructive, has a lower cost, and is easier to operate [17,18]. Computer vision-based marbling analysis procedures involve the segmentation of marbling from the meat images and the characterization of marbling features. Since the segmentation of marbling from the pork image is the basis for assessing marbling characteristics, the accuracy and efficiency of marbling segmentation matter the most.

Manual segmentation of the marbling regions from the pork image is a time-consuming process. Automated marbling segmentation algorithms have been the focus of research, but most algorithms were designed for beef rather than pork [17,19,20,21]. For example, Liu et al. used a hyperspectral imaging technique based on Gabor filters and wide line detectors to identify marbling patterns on a cutting surface of pork [22,23]. Chen et al. quantified the percentage of marbling on meat images using conventional digital image processing techniques [20]. Cernadas et al. designed a machine learning-based marbling segmentation using color texture features [21]. In recent years, images captured by consumer digital cameras have been used for analysis, and support vector machines (SVMs) have been employed as a form of machine learning to segment marbling [24,25]. The disadvantages of these methods mainly focus on using hand-crafted features to obtain the segmentation results. The representative features are sensitive to the change in image background and quality, which reduce model generality and robustness. Therefore, automatic feature extraction methods are urgent and meaningful for marbling segmentation.

With the development of deep learning, convolutional neural networks (CNNs) have attracted huge attention from researchers in image segmentation. Using the hierarchical structure with multiple convolutional neural layers, CNNs can automatically extract the representative features from the input layer by layer and learn the complex data structure in large datasets. Over the past decade, a rich class of fully convolutional networks (FCNs) has been proposed to extend classification convents to segmentation by using a pre-trained CNN as the backbone and fine-tuning fully convolutionally to effectively take the input of any size and accurately predict the outputs of the corresponding sizes [26]. U-Net is one of the most important end-to-end FCNs designed for biomedical image segmentation. It introduces a U-shaped encoder–decoder architecture and long skip connections between the corresponding lower and upper layers to better capture context and enable precise localization [27]. For most biological tasks where thousands of training images are beyond reach, U-Net accomplishes good training from very few images by extensively adopting data augmentation. Therefore, U-Net and its variants have outperformed state-of-the-art biomedical segmentation applications such as cells, neurons, brains, and many other tissues and organs [27,28,29,30]. Zhao et al. attempted the U-Net on beef marbling segmentation [31]. As expected, U-Net achieves a satisfying performance and shows superiority to K-means and OTSU methods in model stability, generalization, and robustness. Unfortunately, to the best of our knowledge, there has yet to be a report using deep learning models to segment pork marbling.

Segmentation of pork marbling is more challenging than beef marbling. The pork marbling objects are much thinner and smaller, occupy a smaller portion of the whole image, are sparsely distributed, and exhibit more considerable scale variations. Other characteristics, e.g., lower color contrast with lean meat and more blurred object boundaries, further impact the final segmentation results. Many approaches have been proposed for small and thin object segmentation. A promising approach is to enlarge the input images to increase the resolution of small and thin objects or construct high-resolution feature maps. However, it will inevitably cost significant GPU consumption to run the network. The size of the enlarged input is limited by the amount of memory available in the GPUs. A patch-based training strategy refers to cropping the image into small patches as the input for training [27,32]. Cropping the whole image into patches and then enlarging the resolution of individual patches seems to be a feasible training mode for addressing the aforementioned problem. It can alleviate the memory consumption caused by using deep learning models to calculate large image inputs and increase the number of samples used for training.

Another widely used method for small and thin object segmentation is to modify the network structure to enhance high-level small-scale features with multiple lower-level feature layers. PSPNet and DeepLab design the spatial pyramid pooling module (PPM) and atrous spatial pyramid pooling (ASPP) [33,34]. DANet employs the self-attention mechanism on top of a dilated FCN [35]. CE-Net proposes a contextual encoding module, which integrates a dense atrous convolution (DAC) block and a residual multicore pooling (RMP) block and arranges them in tandem between the feature encoder and feature decoder blocks [36]. Enhancing multi-scale representation is also an effective way to address objects with large-scale variations in semantic segmentation, which benefits our pork marbling segmentation tasks. Changing the loss function is another effective manner to improve the segmentation accuracy of small and thin objects [37,38]. Since the small and thin objects occupy a small portion of the image, a class imbalance usually exists in the segmentation, causing the failure of the traditional cross-entropy loss function. The addition of weights to every class in the loss equation is commonly utilized to address the class imbalance problem, where pixels of a small proportion are assigned high weights, while pixels of a high proportion are assigned low weights [38]. Since the loss function plays a vital role in optimizing the deep learning model, direct optimization of the evaluation metric of semantic segmentation has also been proved to be applicable to the imbalanced class problem.

In this study, we proposed a patch-based training framework with the lightweight Marbling-Net, aiming to provide an effective and efficient way to obtain accurate marbling segmentation results from the pork images by using a few available training samples. Instead of directly employing the original images as input for training, we broke the pork images into patches and up-sampled and extensively used geometric data augmentations on the patches in order to improve the performance of segmenting small and thin marbling objects, alleviating the large memory usage and making full use of the limited training samples. The Marbling-Net was designed as a lightweight FCN to avoid the loss of small and thin marbling targets during the convolutional and pooling operations. Inspired by CE-Net [36], a context extractor module, including DAC and RMP blocks, was inserted within the encoder–decoder structure to enhance the multi-scale representation of pork marbling. In addition, considering the class imbalance due to the small portion of marbling in the pork image, we adopt Lovász–Softmax loss as a complementary optimization for the intersection over union (IoU) metric. To verify the practicality and effectiveness of the network, we collected and released a pixel-wise annotation marbling dataset, the pork marbling dataset 2023 (PMD2023). Using smartphones, 173 RGB images of pork LD muscles were acquired under real-world slaughterhouse conditions. Experiments on the PMD2023 verify that the proposed method outperforms other state-of-the-art methods. The trained model enables accurate quantification of many image-based pork marbling characteristics, such as the marbling ratio, which can be a critical meat quality trait used by pig breeders and geneticists to conduct genetic improvement research on meat quality.

## 2. Materials and Methods

### 2.1. Pork Marbling Dataset PMD2023

**Figure 1:**
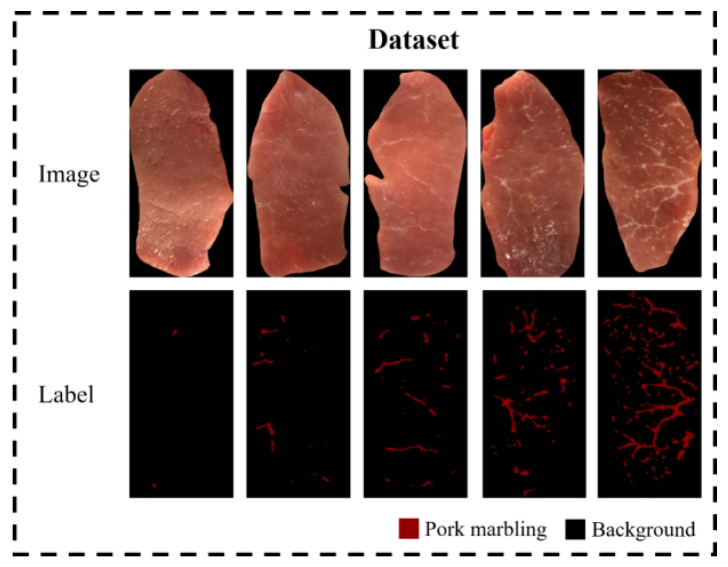
Examples of pork images and annotations of the longissimus dorsi muscle with different marbling content in the PMD2023 dataset.

Since no public dataset was available for pork marbling segmentation, we collected and released the pork marbling dataset (PMD2023). The PMD2023 consists of 173 images of the pork LD muscles with pixel-level annotations. Each image was collected from different pigs. Examples of the pork marbling images and corresponding annotations in the PMD2023 are shown in Figure 1.

#### 2.1.1. Data Collection

Two hundred and fifteen pigs were sampled from an advanced generation intercross population of Large White and Tongcheng pigs. All pigs were raised on the Yunzhi pig farm (Tongcheng County, Xianning, Hubei province, China) with the same feeding and management conditions until slaughtered at 115.80 ± 13.88 kg of live weight. A total of 2.5 cm slices of the LD muscle were dissected from the sternal rib end in the 3rd to 4th rib of the left half of the freshly slaughtered carcass and refrigerated at 4 °C for 24 h. After trimming off the subcutaneous and connective tissues, the slices of the LD muscle were used for image acquisition in a black disk.

The images of pork LD muscle slices were collected using the rear camera of a handheld mobile phone (iPhone 6), which was an 8-megapixel, 1080p “iSight” camera with a pixel size of 1.5 microns and an aperture of f/2.2. The distance between the camera and the slice of pork LD muscle was about 40 cm. The image was saved in JPEG (Joint Photographic Experts Group).

Images were acquired from morning to afternoon (8:00 a.m. to 5:00 p.m.). The illumination in the slaughterhouse was provided by 35-watt LED lights, which were installed every 20 m^2^. Finally, 173 high-quality images dissected from different pigs were selected to construct the PMD2023 dataset.

For each slice of the LD muscle, we measured the IMF content and recorded the marbling score. The measurement procedures of IMF content followed the National Standards “Livestock and poultry meat quality test-Guideline for near-infrared spectroscopy method” (GB/T40467-2021) of the People’s Republic of China. The IMF content of each LD muscle was estimated with a FoodScan^TM^ Meat Analyser (FOSS, Demark). The marbling score was visually graded by two meat quality experts followed by National Pork Producer Council (NPPC) guidelines [39]. The scale of the marbling score ranges from 1 to 10.

#### 2.1.2. Data Annotation

The resolution of the original image was 2448 × 3264 due to the reality that the pork LD region in each image only occupied about 1/6 of the original image area. The pork LD region in each image was cropped by drawing the minimal enclosing rectangle. The obtained pork LD images were uniformly resized to 400 × 800. Pixel-wise annotation of the marbling regions was then performed on each pork image using LabelMe software [40]. As shown in Figure. 1, the marbling regions were labeled as foreground using red color (RGB: 128, 0, 0), and the rest of the regions, including the lean area and the backboard beneath the pork in the images, were all labeled as the background using black color (RGB: 0, 0, 0). All 173 pork images in the PMD2023 were annotated in a fine pixel-wise label style.

#### 2.1.3. Data Preprocessing

Since the surface of the pork in the image is reflective, there are inevitably some reflective spots on the pork images. To reduce the reflective noise and enhance the edge of marbling regions, the mean shift filter operation was applied to the images for both model training and inferencing. The mean shift filtering is a data clustering algorithm widely used to retain the edges and texture in image segmentation tasks [41]. In this study, the mean shift filter operation was conducted by calling the pyrMeanShiftFiltering function in the cv2 library, where the defined shift physical space radius was set to 5 and the shift color space radius was set to 10.

### 2.2. Patch-Based Training Framework for Pork Marbling Segmentation

**Figure 2:**
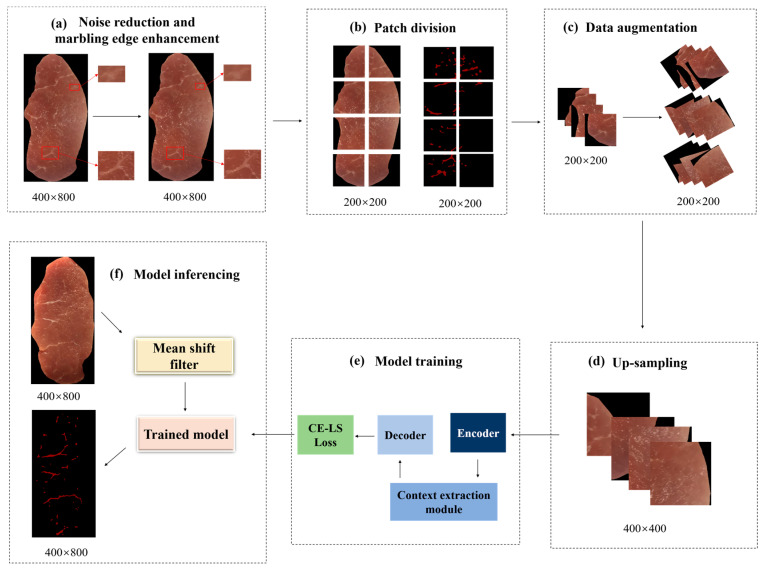
Flowchart of the patch-based framework for marbling segmentation. (**a**) Noise reduction and marbling edge enhancement using mean shift filter operation. (**b**) Patch division. (**c**) Data augmentation. (**d**) Up-sampling. (**e**) Model training. (**f**) Model inferencing.

Pork marbling is formed by intramuscular fat flecks and stripes with slender and irregular shapes, large-scale variations, blurred boundaries, and low color contrast with lean meat. During segmentation, we had to face the challenge of missing thin and small marbling objects, insufficient extraction of multi-scale features, and edge noise. To tackle these problems, a patch-based training framework was proposed for pork marbling segmentation with the Marbling-Net, which took the cropped patches of the pork image as the input and scaled up the input patches to enlarge image resolution, and was designed on the basis of an encoder–decoder structure with the integration of a context extractor module to fully exploit the spatial and semantic information of marbling. The context extractor module comprises the dense atrous convolution (DAC) block and residual multi-kernel pooling (RMP) block to better capture the marbling texture of multi-scale context. A new loss function by combining the cross-entropy loss function and Lovász–Softmax [42] was designed to correct the imbalance class between the marbling and background pixels. Considering that only a few training samples were available, we heavily utilized data augmentation to teach the network the shift and rotation invariance. The overall framework of the patch-based training for marbling segmentation is shown in Figure 2.

#### 2.2.1. Patch-Based Training

In this framework, the cropped patches were only used for model training. At the same time, the inferencing was performed on the entire pork image for the pixel-to-pixel predictions. Therefore, 138 images in the training dataset of the PMD2023 were divided into patches. These patches were generated in a fixed size s×s by sequential slicing from left to right and top to bottom of the pork image without the overlay. We conducted extensive experiments to search for the optimal patch size for training the Marbling-Net on the PMD2023 dataset.

In this study, we investigated the influence of patch size on the model performance. Since the pork image in the PMD2023 was 400 × 800, each image was divided into 128, 32, 8, and 2 patches, respectively, when the patch size was set at 50 × 50, 100 × 100, 200 × 200, and 400 × 400. In total, four patch-based training datasets of different fixed patch sizes were prepared.

#### 2.2.2. Data Augmentation

Data augmentation is essential to train a deep learning-based segmentation network when only a few annotated images are available. It increases the amount and diversity of the annotated training samples to teach the network the desired invariance and robustness properties. Regarding pork images, we primarily needed shift and rotation invariance to enhance model robustness to shooting angles and positions. In this study, a series of online geometric data augmentation was implemented sequentially on the training dataset using an open-source image augmentation library Albumentation [43]. Image transform operations included horizontal flipping with a probability of 0.5, counterclockwise rotating by 90 degrees with a probability of 0.5, random angle rotation (0 ± 180° degrees) with a probability of 0.2, translation (0 ± 0.0625), and scaling (1 ± 0.2). Online data augmentation is a commonly used image augmentation method, which augments data during model training. Since each epoch trains on different images, theoretically, the amount of data during the training process is infinite. We estimated the augmented training dataset, which increased from the original 1104 patches to 92,736 patches if the patch size was set to 200 × 200.

#### 2.2.3. Fully Convolutional Networks for Marbling Segmentation (Marbling-Net)

**Figure 3:**
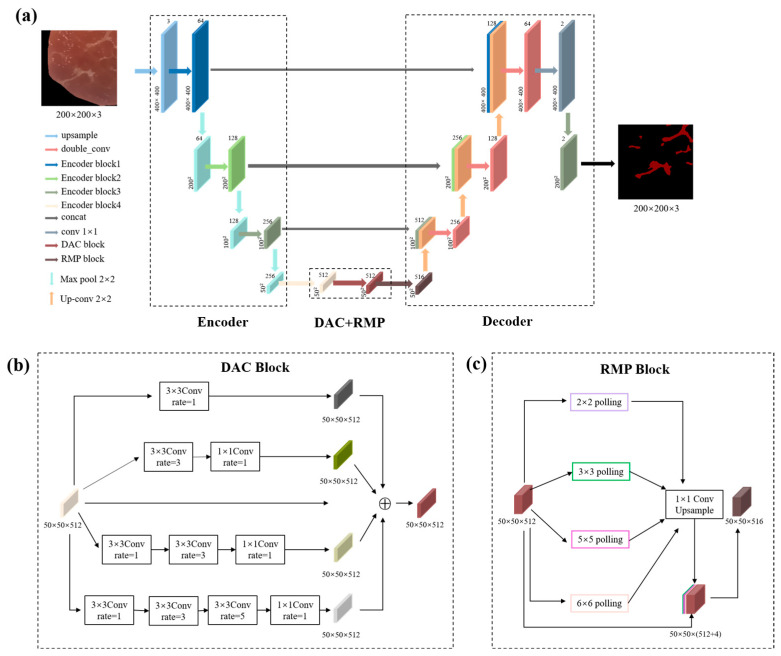
The pipeline architecture of the Marbling-Net. (**a**) The overall architecture of the Marbling-Net. (**b**) The architecture of the dense atrous convolution (DAC) block. (**c**) The architecture of the residual multicore pooling (RMP) block. The fundamental backbone model is a U-Net based on VGG-16. The DAC and RMP blocks incorporated into U-Net at the end of the encoder module are shown in the dashed box at the bottom. Boxes of each color correspond to a multi-channel feature map. The number of channels is indicated at the top of the box. The x-y dimensions are provided at the bottom left edge of the box. Arrows of different colors in the lower left corner of the picture indicate various operations.

Our proposed Marbling-Net was designed on the basis of the FCN. As the network deepens, the convolutional and pooling layers of the FCN will reduce the resolution, causing the loss of small and thin pork marbling. Therefore, the Marbling-Net was modified from the U-Net framework with a shallow VGG-16 as the backbone to effectively capture the low-level spatial information from pork marbling for accurate segmentation. The overall architecture of the Marbling-Net is shown in Figure 3a.

(1) encoder–decoder structure

In the encoder part, we used the first ten layers of VGG-16 as the encoder, which reduced the parameters of the network while ensuring segmentation accuracy. The image patches were the input of the network. They were firstly up-sampled with a bilinear interpolation filter to increase the image resolution and enlarge the image details, facilitating feature extraction of small pork marbling. Then the up-sampled patches sequentially passed through four encoding blocks to extract pyramid features. The four different encoding blocks are the first two layers of VGG-16, the third and fourth layers of VGG-16, the fifth, sixth, and seventh layers of VGG-16, and the eighth, ninth, and tenth layers of VGG-16, respectively. A maximum pooling operation is incorporated after the first three encoding blocks with a stride of 2 and a convolution kernel size of 2 × 2 for down-sampling. The backbone extracts feature maps of five different sizes 400 × 400, 200 × 200, 100 × 100, and 50 × 50. The decoder part of the Marbling-Net follows the typical U-Net architecture, which comprises two successive 3 × 3 convolutions, each followed by a batch normalization (BN) layer and a rectified linear activation (ReLU) function to speed up the convergence of the network. Deconvolution can gradually restore the spatial and edge information in the original image and finally map the low-resolution feature maps to the feature maps of full-input resolution for pixel-level classification.

The encoder–decoder structure can extract high-level context information but also has certain disadvantages. The image is compressed during the encoding process, resulting in the loss of a lot of detailed information, which is critical for accurate image segmentation. Using the skip connection structure, U-Net can fuse low-level and high-level features, enabling the network to retain more high-resolution, detailed information in the encoder and preserve more spatial features in the decoder to promote segmentation accuracy. Hence, we followed the same U-Net strategy that adopts skip connections between the encoder and decoder to extract multi-level features, as shown in Figure 3a.

(2) Multi-scale feature extraction module

The encoder–decoder architecture with skip connections only captures multi-scale features within the limited scaling range. Considering huge differences in the shape and length of different pork marbling, multi-scale features of marbling were needed for accurate segmentation. Inspired by CE-Net [36], a context extraction module consisting of the DAC and RMP blocks was added at the end of the encoder of our Marbling-Net to further extract the multi-scale features of pork marbling. The detailed structure of the context extraction module is described below.

The structure of the DAC block is shown in Figure 3b, which consists of four branches stacked in the atrous convolution in cascade mode. The number of atrous convolutions in the four branches gradually increases from 1 to 1, 3, and 5, and then the receptive field of each branch will be 3, 7, 9, and 19. Therefore, the block can extract features from different scales [36]. A 1 × 1 convolution is applied at the end of each atrous branch for rectified linear activation. Finally, the output features of the DAC block are obtained by directly combining the features before inputting the DAC block with the output features of the four branches. Since the DAC block combines dilated convolutions with different expansion rates, it has different sizes of receptive fields, and other receptive fields have additional extraction capabilities for target features of different sizes. Therefore, the network can better extract pork marbling features of various sizes.

The RMP block is made up of multiple convolution kernels of different sizes for max pooling operations. Therefore, it possesses multiple effective field-of-views and can also benefit the detection of pork marbling in various sizes. As shown in Figure 3c, RMP encodes global context information using four receptive fields of different sizes: 2 × 2, 3 × 3, 5 × 5, and 6 × 6. The outputs of four branches contain feature maps of various sizes. After each max pooling branch, a 1 × 1 convolution is applied to change the number of the feature map channels to 1. Then, the low-dimensional feature map is up-sampled to the same size as the original one by bilinear interpolation. Finally, the original features are concatenated with the up-sampled feature map.

We set $F_{E}(X;\theta_{E},s)$ as the encoder module, where $\theta_{E}$ represents the parameters of the encoder and 𝑠 represents the number of feature maps of the feature pyramid. Then:

(1) $$\left(f_{1},f_{2},f_{3},f_{4}\right)=F_{E}(X;\theta_{E},s)$$

where $f_{1},f_{2},f_{3},f_{4}$ correspond to four feature maps of different sizes extracted by the encoder module. The size of $f_{4}$ is 50 × 50, which is the largest feature map and is the output of the RMP block.

#### 2.2.4. Loss Function

The proposed network aims to predict each pixel as the foreground (marbling) or background (the rest region in the pork image), which is a pixel-wise classification problem. The most common loss function adopted in FCN-based image segmentation methods is the pixel-wise cross-entropy loss designed to optimize the pixel classification accuracy. However, marbling regions occupy a small portion of the whole pork image, leading to the foreground–background class imbalance problem and influencing the segmentation accuracy. The IoU is a vital evaluation metric of semantic segmentation. Direct optimization of the IoU in the training process is an effective measure to deal with the imbalanced class problem and improve the segmentation performance of the Marbling-Net. Therefore, we incorporated the Lovász–Softmax loss as an auxiliary loss to directly optimize the IoU in the training process. Hence, we trained the Marbling-Net with the composite loss function (CE-LS loss, denoted as $L_{ce-ls}$) by a combination of cross-entropy and Lovász–Softmax losses, which is formulated as:

$$L_{ce-ls}=L_{ce}+L_{ls}$$

where $L_{ce}$ represents a pixel-wise cross-entropy loss for optimizing the pixel classification accuracy and $L_{ls}$ represents the Lovász–Softmax loss for optimizing the IoU. The detailed principles of pixel-wise cross-entropy and Lovász–Softmax losses are described as follows.

Cross-entropy loss is a distribution-based loss widely used in classification and segmentation tasks. It measures the difference between GT and predicted distributions. The semantic cross-entropy loss function based on deep learning can be formulated as:

(2) $$L_{ce}(y,p)=-\frac{1}{N}\sum_{n=1}^{N}\sum_{c=1}^{C}y_{n,c}logp_{n,c}$$

where N represents the total number of pixels, C represents the number of categories, $y_{n,c}\in \{0,1\}$ represents the ground-truth, and $p_{n,c}\in \{0,1\}$ represents the predicted pixel category.

The Lovász–Softmax loss directly optimizes the Jaccard index by linear convex surrogate based on the Lovász extension of the submodular set function [42]. The Jaccard index, also known as the intersection over union, is a commonly used evaluation indicator for image segmentation results. Since the Lovász–Softmax is directly optimized on the Jaccard index without paying attention to the number of samples, it can solve the class imbalance problem to a certain extent. The Lovász–Softmax loss function is expressed as:

(3) $$L_{ls}=\frac{1}{\left|c\right|}\sum_{c\in C}{∆}_{{\bar{J}}_{c}}(m(c))$$

where $L_{ls}$ is the Lovász–Softmax loss and c is the category, such as 1, 2. m(c) is the error vector class c, $m\left(c\right)\in {\left\{0,1\right\}}^{p}$ and p is the calculated pixel number, $m_{i}\left(c\right)=\left\{\begin{array}{c} {1-f}_{i}\left(c\right)\;if\;c=y_{i}^{*}\\ f_{i}\left(c\right)\;otherwise \end{array}\right.$, $m_{i}\left(c\right)=\left\{\begin{array}{c} {1-f}_{i}\left(c\right)\;if\;c=y_{i}^{*}\\ f_{i}\left(c\right)\;otherwise \end{array}\right.$, ${∆}_{J_{c}\left(y^{*},\tilde{y}\right)}=1-J_{c}\left(y^{*},\tilde{y}\right)$, ${\tilde{y}}_{i}$ is the GT category of the pixel i, and ${\tilde{y}}_{i}$ is the predicted category. ${∆}_{{\bar{J}}_{1}}$ is the Lovász extension [42] to ${∆}_{J_{1}}=1-J_{1}$.

## 3. Experiments

### 3.1. Implementation Details

The steps and parameters for model training are as follows. To evaluate the stability and reliability of the model in such a small dataset, we conducted five-fold cross-validation on all experiments, where different training and validation sets were used for each of the five cross-validation runs. The 173 pork marbling images were randomly divided into 5 mutually exclusive subsets. There was 1 subset used as the validation set (35 images), and the other 4 subsets were used as the training set (138 images). For each run, images in the training set were first divided into patches, and we conducted an online data augmentation, while the images in the validation set were not divided into patches.

Considering the memory limitation of the GPU, the batch size was set to 4 during training, and the labeled data was processed accordingly. The Adam optimizer was used for training, in which the poly strategy was used for learning rate decay with the initial learning rate of 0.0001, with 400 epochs for training, and the early stopping strategy was used to prevent overfitting. Poly is a strategy of exponential transformation. The specific formula is as follows:

(4) $$lr=base\_lr\times {\left(1-\frac{epoch}{num\_epoch}\right)}^{power}$$

where lr is the new learning rate, base_lr is the benchmark learning rate, epoch is the number of iterations, num_epoch is the maximum number of iterations, and power controls the shape of the curve. Here, we set it to 1.

The hardware for training the Marbling-Net is a GPU server equipped with an Intel Xeon (R) E5-2650 CPU and four GeForce GTX 1080Ti GPUs with 11 G of memory, but only one of the four GPUs was used. The entire training process is implemented using the PyTorch framework running on the CentOS 7.7 operating system.

### 3.2. Evaluation Metrics

We selected the four most commonly used indicators in semantic segmentation as the evaluation criteria for our model, including the IoU, precision, recall, and F1-score.

#### 3.2.1. Intersection over Union

The IoU is one of the most commonly used metrics for image segmentation, defined as the ratio of overlap area to union area between the segmented map and the ground truth. Here, we only compute the IoU for the marbling category in the validation dataset and take the average. The specific calculation formula is as follows:

(5) $$IoU=\frac{N_{TP}}{N_{TP}+N_{FN}+N_{FP}}$$

where $N_{TP}$ is the total number of pixels that are true positives in the validation set. $N_{FN}$ is the total number of false negative pixels in the validation set. $N_{FP}$ is the total number of false positive pixels in the validation set.

#### 3.2.2. Precision

Precision indicates the proportion of samples that are predicted to be correct (ground truth is positive) among all samples predicted by the model as positive examples. The calculation formula is as follows:

(6) $$Precision=\frac{N_{TP}}{N_{TP}+N_{FP}}$$

#### 3.2.3. Recall

Recall is defined as the percentage of all samples with positive labels that are predicted, and the calculation method is as follows:

(7) $$Recall=\frac{N_{TP}}{N_{TP}+N_{FP}}$$

#### 3.2.4. F1-Score

F1-score is the harmonic mean of precision and recall, and its calculation method is as follows:

(8) $$F1-Score=\frac{2*Precision*Recall}{Precision+Recall}$$

## 4. Result

### 4.1. Characterization of the PMD2023 Dataset

**Figure 4:**
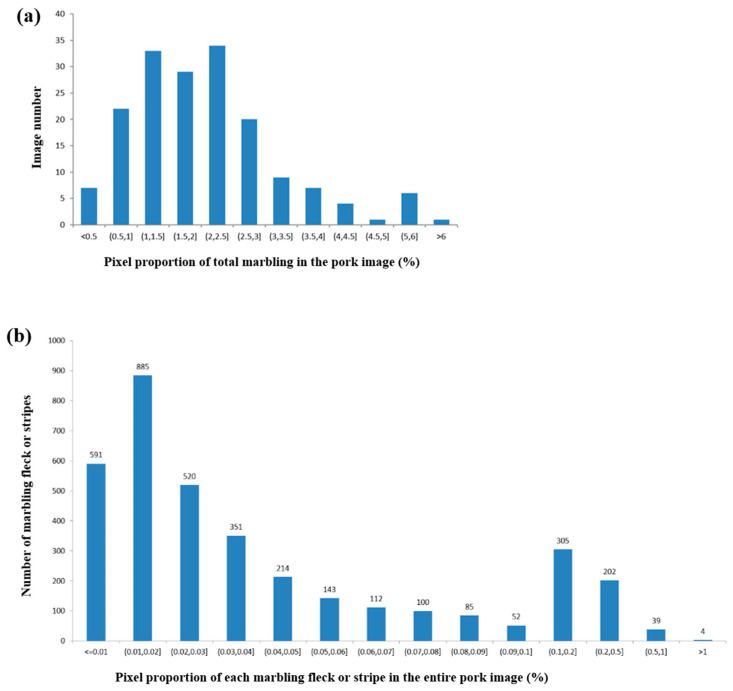
Distributions of the marbling proportion in the pork images of the PMD2023 dataset. (**a**) The distribution of pixel proportions of all the marbling pieces in the pork image. (**b**) The distribution of pixel proportions of each marbling piece or stripe in the pork image.

We generated the PMD2023 dataset and conducted the pixel-wise annotation of each image for the pork marbling segmentation task. Before model construction, we analyzed the characteristics of marbling objects in these pork images. Observing the examples of pork images in Figure 1, marbling appears as small white flecks or thin stripes sparsely distributed within the pink lean. We calculated the pixel proportion of marbling in each image and plotted the distributions of the marbling proportion among the PMD2023 dataset. As shown in Figure 4a, nearly 90% of the images have a marbling proportion of less than 3.5%, and the proportion of marbling in the majority of pork images ranges from 0.5% to 3%. Therefore, pork marbling objects occupy a small proportion of each pork image in PMD2023 datasets.

Pork marbling also exhibits the characteristics of large intra-class scale variation. We calculated the pixel proportion of each marbling fleck or stripe to the entire image in each image and analyzed their distributions across all the images in the PMD2023. It can be seen in Figure 4b that 591 pieces of marbling are super tiny and occupy a pixel proportion of less than 0.01% of the entire image, and 550 pieces are on a relatively large scale with a pixel proportion larger than 0.1%. The largest piece of marbling occupies 8151 pixels, while the smallest one only has 2 pixels, which shows that the marbling class has multi-scale characteristics. In summary, pork marbling in pork images is small and has thin targets with large-scale variation, which brings great challenges for accurate segmentation.

### 4.2. Comparison with Representative Methods

**Figure 5:**
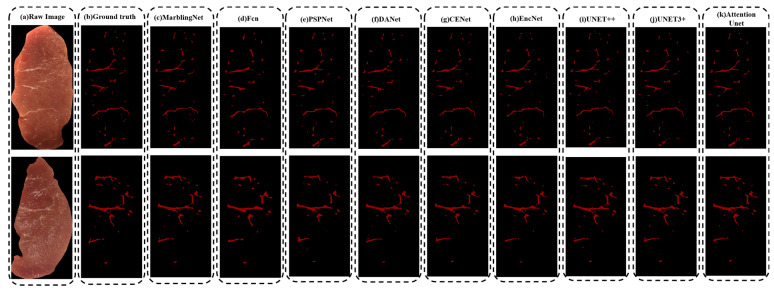
Visual comparison of marbling segmentation results between the Marbling-Net and other methods. (**a**) Raw image. (**b**) Ground truth. (**c**) Marbling-Net. (**d**) FCN. (**e**) PSPNet. (**f**) DANet. (**g**) CENet. (**h**) EncNet. (**i**) UNet++. (**j**) UNet3+. (**k**) Attention U-Net.

We designed the Marbling-Net and patch-based training strategy to precisely segment marbling in pork images. To verify the effectiveness of the proposed method, this section evaluated the segmentation results of our proposed Marbling-Net against other cutting-edge segmentation models. We selected models that perform well in semantic segmentation tasks and variants of U-Net for comparison, including the FCN [35], PSPNet [34], DANet [44], UperNet [45], EncNet [46], CE-Net [36], U-Net++ [30], U-Net3+ [29], and Attention U-Net [28]. For a fair comparison, all experiments had the same training and test samples had the same hyperparameters. The models used for comparison were all based on the patch-free method. To comprehensively compare these models, we evaluated the overall segmentation performance of these methods using precision, recall, F1-score, and the IoU. The accuracy results of training under different models and the Marbling-Net are shown in Table 1. The segmentation results of the different models are exhibited in Figure 5.

As shown in Table 1, the proposed Marbling-Net outperforms the other methods on the PMD2023 dataset. The Marbling-Net surpassed the second-highest Attention U-Net by about 2.1%, 3.1%, and 1.4% in terms of the IoU, recall, and F1-score, respectively. The Marbling-Net had a significantly improved recall compared to the U-Net and its variants, but its precision advantage has yet to become apparent. By observing the visualization results of different models in Figure 5, we can further demonstrate the benefits of the proposed Marbling-Net. Compared to other methods, the Marbling-Net can delicately extract more small marbling flecks and precisely capture the slender marbling areas with better completeness and clearer edges. In summary, the proposed Marbling-Net can better capture the marbling of different scales and shapes in the image and segment these marbling areas more completely.

The model parameter size and inference time for a single image were also compared, and the results are shown in Table 1. In terms of model parameters, the Marbling-Net has relatively fewer parameters. However, introducing the up-sampling operation can lead to a longer inference time for the Marbling-Net. Nevertheless, the time cost of 249.81 ms per image (in other words, four images per second) is acceptable for the majority of practical applications when measuring marbling traits. When used to analyze a single image, it does not cause users to feel severe lag. Sacrificing some inference time to improve segmentation accuracy is necessary, as accuracy matters the most for the accurate quantification of marbling traits.

### 4.3. Ablation Study

In order to explore the effectiveness of the patch-based training, up-sampling operation, and the context extraction module in the proposed marbling segmentation framework, we conducted a number of ablation experiments on the PMD2023 dataset. Since the proposed Marbling-Net was based on the U-Net, we set the U-Net as the baseline model. As shown in Table 2, applying patch-based training, up-sampling operation, and the context extraction module could improve the marbling segmentation performance, especially the patch-based training strategy, which contributed the most. When only the patch-based training is adopted, the evaluation metrics of the IoU, precision, recall, and F1-score increased by 2.1%, 0.7%, 2.0%, and 1.4%, respectively, compared to the baseline. The context extraction module can slightly improve performance. Compared to the baseline model, only adopting the context extraction module can increase the IoU, recall, and F1-score by 0.4%, 0.8%, and 0.3%, respectively. These results illustrated the significance of our introduction of the context extraction module.

Up-sampling the patch before inputting it into the network can increase the image resolution of the input, which may benefit learning the representative features of small targets. To verify this assumption, we also conducted ablation experiments. As shown in Table 2, the network, by addition of both the up-sampling of patches and the context extraction module, improved the IoU, precision, recall, and F1-score by 0.8%, 0.8%, 0.3%, and 0.5% compared to the network only using the patch-based training. However, when only the patch-based training was added without adding the context extraction module, the prediction results were not better than the patch-based training alone. It is because only an up-sampling patch led to the model insufficiently learning the marbling features, while adding a context extraction module on the basis of an up-sampling patch can greatly enhance the feature extraction ability of the network. This also confirmed the effectiveness of the context extraction module added to the network.

### 4.4. Comparison of Loss Functions

**Figure 6:**
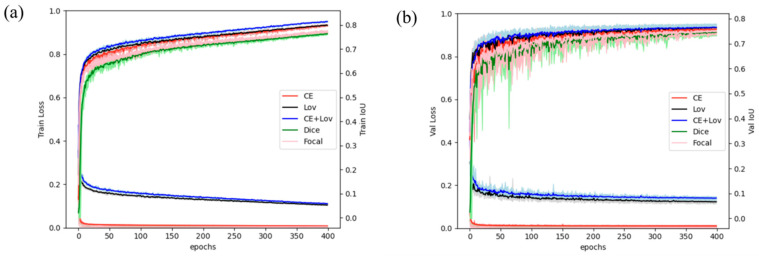
The performance of the Marbling-Net using five different loss functions across 400 epochs under five-fold cross-validation. (**a**) Training curves; (**b**) validation curves. Bold lines denote the averaged values of loss and the MioU, and the translucent bands denote the range of loss and the MIoU across five folds. Red, black, blue, green, and pink lines denote the network results using the cross-entropy (CE) loss, Lovász–Softmax (LS) loss, the combination of cross-entropy and Lovász–Softmax (CE-LS), dice loss, and focal loss, respectively.

The loss function plays a critical role in the optimization of the deep learning network. For the task of segmentation, the choice of loss function determines the speed of model converges and the accuracy of the model to some extent. Therefore, we first conducted experiments to demonstrate the effectiveness of the CE-LS loss adopted on the Marbling-Net. In addition to CE-LS loss, four other commonly used semantic segmentation losses, dice loss [47], focal loss [48], cross-entropy loss [49], and Lovász–Softmax loss [42], are attempted on the Marbling-Net. The IoU curves of the training and validation process for the marbling category using different loss functions are shown in Figure 6. During the entire convergence process, the IoU fluctuates upward as a whole, while the loss fluctuates downward, indicating that the model was constantly learning rather than falling into local optimization. In addition, it can be seen in Figure 6b that the curve of the CE-LS loss function was smoother during the training phase, indicating that the CE-LS loss was more conducive to network convergence and the training was more stable. We compared the segmentation performance of the Marbling-Net on five loss functions according to the IoU of the marbling category. As shown in Table 3, the model using LS-CE loss achieved the highest segmentation performance, which was 0.6%, 1.4%, and 0.4% higher than using cross-entropy loss and 0.3%, 0.2%, 0.2%, and 0.2% higher than using the Lovász–Softmax loss on the IoU, precision, recall, and F1-score, respectively. It validated the effectiveness of our constructed loss function for optimizing marbling segmentation.

We incorporated the Lovász–Softmax to cross-entropy loss with the aim of further optimizing the IoU metric to improve object segmentation, especially for small objects. To explore the optimal weight of the Lovász–Softmax loss in the composite loss function on the Marbling-Net, we set the λ from 0 to 1 for comparative experiments. As shown in Table 4, no significant differences were detected using different λ. However, λ equal to 1 brought the best model performance. Therefore, we set λ to 1, which was the direct addition of cross-entropy and Lovász–Softmax losses.

### 4.5. Comparison of Different Patch Sizes

As the ablation study illustrated, applying patch-based samples to train the network is the critical strategy to improve the performance of marbling segmentation. To study the impact of patch size on the segmentation performance, we experimented with different patch sizes on the PMD2023 dataset. The patch size was set at 50 × 50, 100 × 100, 200 × 200, and 400 × 400, respectively. Considering that the pork LD slice image in the PMD2023 is 400 × 800, each can be divided into 128, 32, 8, and 2 patches without overlay, respectively. Four patch datasets in different patch sizes were prepared for training. As shown in Table 5, the overall segmentation performance of the Marble-Net improved as the patch size increased from 50 × 50 to 200 × 200, but it dropped when the patch size exceeded 200. These results verified that patch size has an impact on the accuracy of marbling segmentation. Therefore, we set the patch size to 200 × 200 for training the Marbling-Net.

### 4.6. Performance Evaluation of Marbling Features Measured by the Marbling-Net

**Figure 7:**
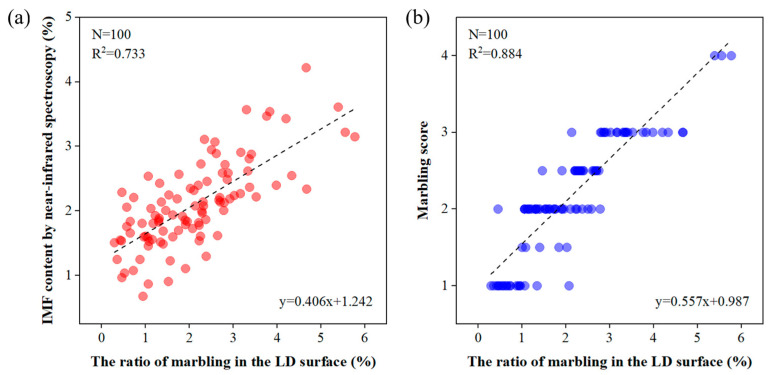
Comparisons between the ratio of marbling in the LD surface measured by the Marbling-Net and the other marbling measurements. (**a**) IMF content measured by near-infrared spectroscopy; (**b**) marbling score by visual grading. Each point represents an individual pork image, $R^{2}$ represents the coefficient of determination, and N represents the number of observations.

We used the segmentation results of the proposed Marbling-Net model to estimate the ratio of marbling in the LD surface by dividing the pixel numbers of marbling by the total pixel numbers of the pork LD muscle. In the experiment, the ratios of marbling in 100 pork LD images selected from our PMD2023 dataset were calculated. These results were compared with the corresponding IMF contents measured by the near-infrared spectroscopy method and marbling scores graded by meat quality experts. A simple linear regression analysis was conducted, and the results are shown in Figure 7. $R^{2}$ for the IMF content and the marbling score were 0.733 and 0.884, respectively, illustrating the reliability of our Marbling-Net model in the analysis of pork marbling features.

## 5. Discussion

Meat quality traits are important economic traits in modern pig breeding programs around the world. IMF contributes importantly to meat quality, which can directly affect the flavor, tenderness, and palatability of meat. Pork marbling is always the representation of IMF, which is highly correlated with IMF contents and can be assessed with eye- or image-based methods. Therefore, marbling characteristics are important meat quality traits for genomic evaluation and the development of molecular breeding markers.

Accurate identification and segmentation of pork marbling in the image is an important step in the quantification of the distribution and amount of marbling characteristics. Although the pixels-to-pixels FCN-based methods have significantly improved segmentation accuracy in large objects, small/thin objects of pork marbling still need to be more challenging to segment due to convolutional and pooling operations that result in information loss.

As shown in Table 2, the proposed Marbling-Net used the patch-based and up-sampling training strategy and was superior to the Marbling-Net trained by the patch-free one in all indicators. Patch-based and patch-free training are two different training modes. Their main difference is whether to divide the input image into several small patches. Patch-based training will break the image into multiple sub-regions (patches) that are input into the network and output prediction results. It is an effective method for small object detection and segmentation. For example, Meng et al. [50] utilized the patch-based training strategy to improve the performance of the CNN-based model for detecting small traffic signs from large images under real-world conditions. Zhang et al. [32] applied patch-based training for remote sensing image semantic segmentation. Patch-based training has the advantages of reducing memory consumption and calculation, fully utilizing the local feature extraction ability of the CNN and increasing the size of the training dataset. However, it also has disadvantages. Patch-based training may lead to information loss or incompleteness. Since each patch only represents a local feature of the image, it ignores the overall context information, which may reduce the generalization ability of the network. However, the segmentation of pork marbling does not depend on the overall context information of the image, but on the quality of the local feature extraction of pork marbling. In addition, considering that the small and thin marbling targets are easy to delete with the pooling operation of the FCN, up-sampling can introduce additional information or constraints, which can enhance the perception of feature details by the FCN. Therefore, the patch-based training strategy together with the up-sampling greatly helps the pork marbling segmentation task.

The choice of the number of backbone layers is of great significance for specific segmentation tasks. A deep FCN is suitable for segmentation tasks in complex scenes [51]. In contrast, our pork marbling segmentation task has a single scene but is filled with many small objects. A deep FCN will cause the loss of some or all feature information of small objects and is easy to overfit, thus affecting the accuracy of network segmentation. Hence, in the encoder part, we used the first ten layers of VGG-16 as the backbone of the Marbling-Net. In addition, we also had to face the segmentation difficulty brought about by the multi-scale features of pork marbling. It is feasible to add a multi-scale feature extraction module at a suitable position in the network. DAC and RMP are combined to form a powerful context extractor that can efficiently extract features of multi-scale objects. We conducted experiments on whether to add multi-scale feature extraction modules on the PMD2023 to prove the effectiveness of the added multi-scale feature extraction modules. The ablation test in Table 2 shows that the segmentation results with the multi-scale feature extraction module added were significantly better than those without the multi-scale feature extraction module.

For the design of the loss function, we investigated the impact of various loss functions on the performance of the marbling segmentation task. Cross-entropy loss is commonly used in most semantic segmentation scenarios and can optimize pixel classification loss to stabilize the training process. However, it has a significant drawback: when the image segmentation task only requires foreground and background segmentation and the number of foreground pixels is much smaller than that of background pixels, the component of y = 0 in the loss function will dominate, causing the model to be heavily biased toward the background, leading to poor performance. Compared to cross-entropy loss, dice loss can handle foreground–background pixel imbalance [47]. Lovász–Softmax loss has been proposed for multi-class semantic segmentation [42]. It integrates a softmax operation into the Lovász loss function, directly optimizing the IoU metric to improve object segmentation, especially for small objects. As shown in Figure 6 and Table 3, adding LS loss to CE loss compensates for the disadvantage of CE loss being affected by class imbalance and enables a smoother training process and more accurate segmentation of pork marbling.

Although our Marbling-Net improved the segmentation quality of pork marbling, it also had a longer inference time. Compared to the other state-of-the-art segmentation models, the Marbling-Net is the slowest. This is mainly due to the integration of the up-sampling operation into the network architecture before the encoder. Without the up-sampling, the inference time of the Marbling-Net would significantly decrease from 249.81 ms to 65.3 ms. However, inevitably, it also affects the segmentation quality. Therefore, more efforts are needed to develop a novel pork marbling segmentation pipeline to improve segmentation performance and, at the same time, reduce the computational time in the future.

## 6. Conclusions

In this article, we proposed a novel training framework for accurately segmenting pork marbling. Considering the small, thin, and multi-scale nature of pork marbling, we made improvements in three aspects: network structure, training constraints, and loss function. Our framework employed a patch-based and up-sampled training strategy, which accommodated large-scale pork marbling and enabled the FCN to pay more attention to local features but also expanded the dataset and mitigated the problem of limited hardware resources. We selected a shallow encoding structure to reduce network parameters while maintaining segmentation accuracy and incorporated a context extraction module at the encoder end to encode features of pork marbling at different scales, capturing more abstract features and preserving more spatial information, thereby enhancing the segmentation performance of pork marbling. To address the sample imbalance between categories, we combined the cross-entropy loss function with the Lovász–Softmax loss to formulate a new loss function (CE-LS loss) that improved segmentation prediction. Our comprehensive experiments on the newly established PMD2023 dataset demonstrated that the Marbling-Net outperforms other state-of-the-art methods and yields more precise segmentation results of pork marbling. The high correlations between the ratio of marbling and the IMF content and the marbling scores illustrated the reliability of the Marbling-Net model in the analysis of pork marbling features.

In conclusion, we give insights on tackling the challenges in pork marbling segmentation. The proposed framework not only provides accurate segmentation results of pork marbling images but also can be further developed as a valuable tool for effectively and efficiently extracting multiple marbling traits from each image. This type of phenotyping tool will facilitate pig breeders to collect pork marbling traits from a pig population, which can be used in the improvement of pork quality.

## Figures and Tables

**Table 1:** Accuracy comparison between the Marbling-Net and other methods.

Method	IoU (%)	Precision (%)	Recall (%)	F1-Score (%)	Parameter (M)	Inference Time (ms)
FCN	31.4	63.7	38.5	46.2	134.3	47.37
PSPNet	54.3	75.0	66.5	67.0	51.4	30.30
DANet	58.5	76.6	71.2	73.8	47.6	60.52
UperNet	64.6	81.4	75.8	76.6	101.4	**15.49**
CENet	65.4	82.3	76.2	79.1	29.0	19.66
EncNet	68.7	84.8	78.3	81.4	34.7	115.44
U-Net	74.3	87.6	83.1	85.2	31.0	53.77
U-Net++	73.7	86.7	83.0	84.8	**9.2**	49.59
U-Net3+	70.1	88.2	77.4	82.4	27.0	169.18
Attention U-Net	74.7	**88.3**	82.9	85.5	34.9	71.29
U-Net (VGG-16)	73.6	87.3	82.5	84.8	41.9	62.40
Marbling-Net (Ours)	**76.8**	87.8	**86.0**	**86.9**	31.0	249.81

**Table 2:** Performance comparison of the Marbling-Net with different components.

Component			IoU (%)	Precision (%)	Recall (%)	F1-Score (%)
Patch-Based	Up-Sampling	Context Extraction Module				
×	×	×	73.9	86.3	83.7	85.0
×	×	√	74.3	86.1	84.5	85.3
√	×	×	76.0	87.0	85.7	86.4
√	×	√	76.1	86.9	85.9	86.4
√	√	×	76.0	87.7	85.0	86.4
√	√	√	**76.8**	**87.8**	**86.0**	**86.9**

**Table 3:** Performance comparison of the Marbling-Net using different loss functions.

Loss Function	Evaluation Metrics			
	IoU (%)	Precision (%)	Recall (%)	F1-Score (%)
Dice	74.7	88.1	83.1	85.5
Focal	75.0	**88.8**	82.8	86.7
CE	76.2	88.5	84.6	86.5
LS	76.5	87.6	85.8	86.7
CE-LS	**76.8**	87.8	**86.0**	**86.9**

**Table 4:** Comparison of the impact of the weight of the Lovász–Softmax loss.

*λ*	0	0.05	0.1	0.2	0.3	0.4	0.5	1
IoU (%)	76.2	76.2	76.5	76.5	76.5	76.7	76.5	**76.8**
Precision (%)	**88.5**	88.3	88.3	87.7	87.5	87.3	87.8	87.8
Recall (%)	84.6	84.8	85.2	85.6	86.0	86.5	85.6	**86.0**
F1-score (%)	86.5	86.5	86.7	86.7	86.7	86.8	86.7	**86.9**

**Table 5:** Performance accuracy comparison of the Marbling-Net using different patch sizes for training.

Patch Size	MIoU (%)	Precision (%)	Recall (%)	F1-Score (%)
50 × 50	73.7	85.0	84.8	84.8
100 × 100	76.2	87.7	85.4	86.5
200 × 200	**76.8**	87.8	**86.0**	**86.9**
400 × 400	76.3	**87.9**	85.2	86.5

## Data Availability

The PMD2023 is available at https://github.com/WeizhenLiuBioinform/Pork_Marbling_Segmentation/tree/master/PMD2023 (accessed on 25 May 2023).
